# Public Pharmacists' Bohemian Concert

**Published:** 1914-05-09

**Authors:** 


### Public Pharmacists' Bohemian Concert.

The Public Pharmacists' and Dispensers' Association had a reunion of members and their friends at Anderton's Hotel, Fleet Street, E.C., last week. Mr. Jas. Hassall France, M.P.S., chairman of the Association, was in the chair, supported by Mr. T. H. W. Idris, J.P., president of the Association, Dr. Churchill Sibley, Dr. Jas. E. Coulson, Mr. R. W. Lindsey and Miss Lindsey, Mr. W. E. Kinsman, Mr. A. Howell, Mr. A. Jenkin, Mr. H. Hewitt, Mr. H. C. Thomas, and their parties, Mr. Geo. W. Gibson, hon. secretary, and party, Mr. A. J. Gibbons, Mr. P. Chaplin, Mr. R. Welford, and Mr. T. S. Goodall and Mrs. Goodall.

During an interval in the proceedings the President expressed on behalf of the members their hearty appreciation of all the good work the Council of the Association had done and was continuing to do to improve the status of the pharmacist engaged in institutional work, especially mentioning Mr. France, chairman, and the secretaries, Mr. Geo. W. Gibson and Mr. R. W. Lindsey. Mr. Idris emphasised the advantage of pharmacists keeping themselves up to date in modern methods of treatment. In this connection the last session's lectures and demonstrations was a sign that the Association's officers do all in their power to arrange the most practical and interesting subjects.

Mr. R. Welford, M.P.S., who is an institutional pharmacist of some thirty-five years' experience, and one of the original founders of the Association, proposed the health of the Chairman. Mr. France, in reply, mentioned the interesting fact that the Association is now working hard to obtain a representative, in the person of Mr. Herbert Skinner, on the Pharmaceutical Council of Great Britain. The election takes place on May 20, when it is hoped that the return of Mr. Skinner will be secured. It is interesting to note that of those who took part in the concert Dr. Coulson, Mr. Harry Graves, and Mr. Leonard Middleton are all engaged in institutional work.

